# *Plasmodium*-induced disruption of brain endothelial barrier integrity in vitro and in mice is prevented by inhibitors of farnesyltransferase

**DOI:** 10.1186/s12936-026-05981-2

**Published:** 2026-06-23

**Authors:** Cláudia Gomes, Kelly A. Crotty, Mariana C. Borges Silva, Marilyn Vasquez, Lizeth Chicas, Isaac Salzano, Ana Rodriguez

**Affiliations:** Department of Microbiology, New York University Grossman School of Medicine, New York, NY USA

**Keywords:** Cerebral malaria, Blood–brain barrier, *Plasmodium falciparum*, *Plasmodium berghei*, Endothelial barrier integrity, Isoprenoids, Farnesyl PYROPHOSPHATE, Farnesyltransferase, Geranylgeranyltransferase, Tipifarnib

## Abstract

**Background:**

Cerebral malaria is a complication of *Plasmodium falciparum* infections where the integrity of the blood–brain barrier (BBB) is compromised, frequently leading to persistent neurological sequelae or death. The current treatment for cerebral malaria is based on rapid elimination of the parasite with intravenous anti-malarial drugs, but there is no adjunctive treatment that could address the loss of BBB integrity, potentially decreasing sequelae and improving the survival rate. Prenylation, a cellular process that incorporates isoprenoid lipids to specific protein sites, is known to regulate endothelial barrier integrity. Here we have studied the role of the isoprenoid farnesyl pyrophosphate (farnesyl-PP) and of different inhibitors of the enzymes that perform prenylation in endothelial cells to determine their effect on the endothelial barrier disruption induced by *Plasmodium falciparum*.

**Methods:**

An in vitro model using human brain microvascular endothelial cells (HBMECs) incubated with lysates of *P. falciparum*-infected red blood cells (iRBCs) and a mouse model of cerebral malaria were used to study the role of the two main prenylation enzymes, farnesyltransferase and geranylgeranyltransferase, in the regulation of endothelial barrier integrity in relation to cerebral malaria.

**Results:**

Addition of exogenous farnesyl-PP to HBMECs incubated with *P. falciparum*-iRBCs resulted in increased endothelial barrier disruption. Conversely, treatment with inhibitors of farnesyltransferase, but not of geranylgeranyltransferase, resulted in protection of barrier integrity against disruption induced by *P. falciparum-*iRBCs. Mice infected with *Plasmodium berghei* that were treated with the farnesyltransferase inhibitor tipifarnib presented delayed onset of cerebral malaria and higher survival rates.

**Conclusions:**

Inhibitors of farnesyltransferase protect human endothelial cell barrier integrity from disruption induced by *P. falciparum* in vitro and decrease mortality in mice with experimental cerebral malaria, indicating a role for farnesylation in the regulation of barrier integrity during cerebral malaria and identifying a potential drug target for the protection of the endothelium during severe malaria.

## Background

Malaria is still a major cause of death in developing countries, especially in African children under the age of five [1]. Cerebral malaria is a serious complication of malaria, that occurs when *Plasmodium falciparum*-infected red blood cells (iRBCs) sequester in brain capillaries, causing impeded blood flow followed by the loss of blood–brain barrier (BBB) integrity, brain swelling, and frequently death [2]. Once neurological symptoms have appeared, a high proportion of patients succumb to cerebral malaria (15–20%), and survivors frequently present permanent neurological sequelae including cognitive dysfunction [3].

Since no specific treatment is available for cerebral malaria, current therapies remain limited to classical anti-malarial treatments that kill the parasite but do not alleviate the endothelial dysfunction that causes this fatal complication [4]. Clinicians agree on the need for adjunctive therapy to save patients from death and/or cognitive impairments, while anti-parasitic therapy eliminates the *Plasmodium* parasite [5].

Upon completion of their infection cycle, *P. falciparum*-iRBCs rupture and release their contents in the circulation. Within the densely packed microenvironment of brain capillaries where iRBCs sequester during cerebral malaria, the iRBC-released contents remain confined in this space [6, 7]. This microenvironment is modeled in vitro by incubating *P. falciparum*-iRBCs over monolayers of human brain microvascular endothelial cells (HBMECs), where effective disruption of the endothelial barrier is observed after rupture and release of the iRBC contents over the endothelial monolayer [8]. Similar endothelial barrier disruption was observed using lysates of iRBCs in the schizont stage (iRBCL) [9].

Endothelial barrier integrity is regulated by multiple intracellular pathways involving Rho GTPases and the actin cytoskeleton, which promote the formation of gaps between endothelial cells, leading to loss of barrier integrity and leakage of plasma [10]. Rho proteins are regulated by prenylation, a post-translational modification that covalently attaches specific isoprenoid lipids (farnesyl and geranylgeranyl) to these proteins, resulting in their insertion in the plasma membrane and subsequent signaling events that culminate in the de-stabilization of intercellular junctions [11].

Here, we have used in vitro cultures of HBMECs incubated with *P. falciparum*-iRBCs and a mouse model of cerebral malaria to study the role of prenylation in the disruption of the BBB during cerebral malaria. We show that addition of farnesyl-PP results in increased endothelial disruption by *P. falciparum*-iRBCs and that inhibition of the enzyme farnesyltransferase, which covalently links farnesyl to target proteins, results in complete protection of endothelial barrier integrity in vitro from *P. falciparum*-iRBCs and partial protection from experimental cerebral malaria in mice. In contrast, inhibition of the related enzyme geranylgeranyltransferase, which transfers the geranylgeranyl isoprenoid to target proteins did not result in endothelial barrier protection. These results provide insight into the regulation of endothelial barrier integrity leading to protection against *P. falciparum*-induced disruption, identifying a potential drug target for the protection of the endothelium during severe malaria.

## Methods

### Culture of endothelial cells

HBMECs from ScienCell were immortalized as previously described [8] and used up to passage 17 [8], primary HBMECs from Cell Systems (ACBRI 376 V) were used up to passage 13, and HPMECs were immortalized as previously described [12] and used up to passage 14. The cells were maintained in Endothelial Cell Media (ScienCell 1001) supplemented with 5% FBS (ScienCell 0025), 1% endothelial cell growth supplement (ScienCell 1052), and 1% penicillin/streptomycin solution (ScienCell 0503). Experiments were performed in Endothelial Cell Media supplemented with 0.5% FBS, 0.1% endothelial cell growth supplement, and 0.1% penicillin/streptomycin solution. The cells were maintained at 37 ºC in 5% CO_2_, grown to ~ 95% confluency then seeded for experiments, and were confirmed mycoplasma-free using the MycoAlert Mycoplasma Detection Kit (Lonza LT07-418).

### Culture, isolation, and lysate preparation of *Plasmodium falciparum*

*P. falciparum* 3D7 was maintained in human RBCs from healthy donors (Grifols, Vista, CA) at 5% hematocrit in RPMI 1640 (Corning 10–040-CV) supplemented with 25 mM HEPES (Fisher Scientific BP310), 25 mM sodium bicarbonate (Sigma-Aldrich S5761), 0.5 mM hypoxanthine (Sigma-Aldrich H9377), 0.5% Albumax II (Thermo Fisher 11021045), and 50 µg/mL gentamicin (Thermo Fisher 15750078). Mycoplasma-free parasite cultures were maintained at 37 ºC in a gas mixture of 5% O_2_, 5% CO_2_, and 90% N_2_, and were synchronized with 5% sorbitol (Sigma-Aldrich S1876) in water (Sigma W3500). Schizont-stage iRBCs were isolated from synchronous cultures using magnetic columns (Miltenyi Biotec 130-042-901). *P. falciparum*-iRBCL and RBCL were generated by freeze-thawing isolated iRBCs or donor-matched RBCs 10 times in liquid nitrogen and a 37 ºC water bath. iRBCL or RBCL were used at a concentration of 8 × 10^6^ (i)RBCs/cm^2^, which corresponds to two densely packed layers of iRBCs over the monolayer of HBMECs as previously described [9].

### Measurement of barrier integrity

Barrier integrity was measured as electrical impedance by the xCELLigence RTCA DP (Agilent, Santa Clara, CA) at 37 ºC and in 5% CO_2_. 12,500 HPMECs or 15,000 immortalized or primary HBMECs were seeded on collagen-coated (40 µg/mL, Sigma-Aldrich C3867) RTCA PET E-Plate VIEW 16 wells (Agilent 300600880) and grown until the cells reached a plateau, after approximately 18–26 h, with impedance measurements taken every 3–6 h. The media was changed to the low-serum media described above and the experimental conditions were added to the cells. Impedance measurements were taken every 15 min over 24 h. The cell index value at the final time point of the growth curve for each well was used to normalize cell index values throughout the 24 h of the experiment [9].

### Fluorescence microscopy

12,500 immortalized HBMECs were grown in collagen-coated, glass-bottom plates (Greiner Bio-One 655981) for 24 h at 37 ºC. Indicated conditions were added to the plate and incubated for 4 h at 37 ºC. Cells were washed 2 × with warm colorless Endothelial Cell Media (ScienCell 1001-b-prf), fixed with 4% paraformaldehyde (Santa Cruz Biotechnology CAS 30525-89-4) for 10 min at RT, and washed 2 × with PBS. HBMECs were stained with phalloidin (1:500 in PBS, Thermo Fisher A12381) for 15 min at RT, washed 3 × with PBS, stained with Hoechst (1:10,000 in Milli-Q water, Thermo Scientific H3570) for 5 min at RT, and washed 3 × with PBS. The cells were imaged with the Olympus IX70 inverted microscope (Evident Scientific, Waltham, MA) using a 60x/1.4 objective (Olympus 1-UB933) and MetaMorph Advanced software version 7.6.5.0 (Molecular Devices, San Jose, CA).

### Experimental conditions

25 µM or 50 µM farnesyl-PP (Isoprenoids FPP003) were added to a monolayer of HBMECs 5 min before the iRBCL or RBCL. 5 µM or 10 µM FGTI-2734 (MedChem Express HY-128350), 10 µM GGTI-2133 (Sigma-Aldrich G5294), 50 µM GGTI-2418 (Cayman Chemical Company 28714), 50 µM FTI 277 (Selleck Chemicals S7465), 5 µM lonafarnib (Sigma-Aldrich SML1457), or 1 µM tipifarnib (Sigma-Aldrich SML1668) were added to the endothelial cells for 1 h at 37 ºC before adding the indicated stimuli of either RBCL, iRBCL, 1U/mL of thrombin (Sigma-Aldrich T6884-100UN), or 25 µM of 18:1 lysophosphatidic acid (Sigma-Aldrich L7260). A summary of the compounds used and their targeted pathway can be found in Table 1. Toxicity to the endothelial cells was determined by xCELLigence, where a toxic decrease was considered following a decrease of barrier integrity of at least 10% in the control conditions (RBCL in combination with the specific inhibitors) compared to RBCL alone. Only non-toxic concentrations were used in the experiments. All inhibitors were used at concentrations known to be active in human cells, as previously described [13–18].

**Table 1:** Summary of compounds and their targeted pathways

Compound name	Pathway of inhibition
FGTI-2734	Farnesyltransferase and Geranylgeranyltransferase
GGTI-2133	Geranylgeranyltransferase
GGTI-2418	Geranylgeranyltransferase
FTI 277	Farnesyltransferase
Lonafarnib	Farnesyltransferase
Tipifarnib	Farnesyltransferase

### Mice

The experimental cerebral malaria model was performed using 4–6-week-old female C57BL/6J mice from Taconic Biosciences (Germantown, NY) that were infected i.p. with 3.5 × 10^5^ *P. berghei* ANKA-infected erythrocytes. Mice were treated with 200µL of vehicle (0.5% (hydroxypropyl)methylcellulose (Sigma-Aldrich H7509) + 0.4% Tween 80 (Sigma-Aldrich P8074) in Milli-Q water) or 100 mg/kg tipifarnib (MedChem Express HY-10502) twice daily by oral gavage [19] from the day before infection with *P. berghei* until the end of the experiment. Parasitemia was quantified by thin blood smear stained with Giemsa (Sigma-Aldrich GS1L), after counting a minimum of 300 RBCs per smear. Mice were monitored twice daily for cerebral malaria signs and were scored as previously described [8]. In brief, mice were scored based on appearance (0 = normal, 1 = ruffled coat, 2 = coat starring or panting) and neurological signs (0 = normal, 1 = hunched, 2 = partial paralysis, 3 = convulsions). Mice were euthanized when presenting with a cumulative score of at least 4. Surviving mice were euthanized on day 9.

### Statistics

Statistical analyses were performed with GraphPad Prism 10 (GraphPad Software, Boston, MA). Normality was first determined with the Shapiro-Wilke test. Data of normal distribution was analyzed by one-way ANOVA with Tukey’s multiple comparisons test or unpaired t-test. Data of non-normal distribution was analyzed by Kruskal–Wallis test with Dunn’s multiple comparisons test or Mann–Whitney test. Logrank (Mantel-Cox) test was used to determine differences in survival. The statistical test used for each graph is denoted in the figure legend.

## Results

### Exogenous farnesyl pyrophosphate enhances the disruption of brain endothelial cell barrier integrity by *P. falciparum*

To investigate the effect of prenylation in barrier integrity of HBMECs, we have used an in vitro system modeling the microenvironment of brain capillaries during cerebral malaria [8], where blood flow is impeded and *P. falciparum*-iRBCs release their contents upon completion of their replicative cycle [6, 7].

**Fig. 1:**
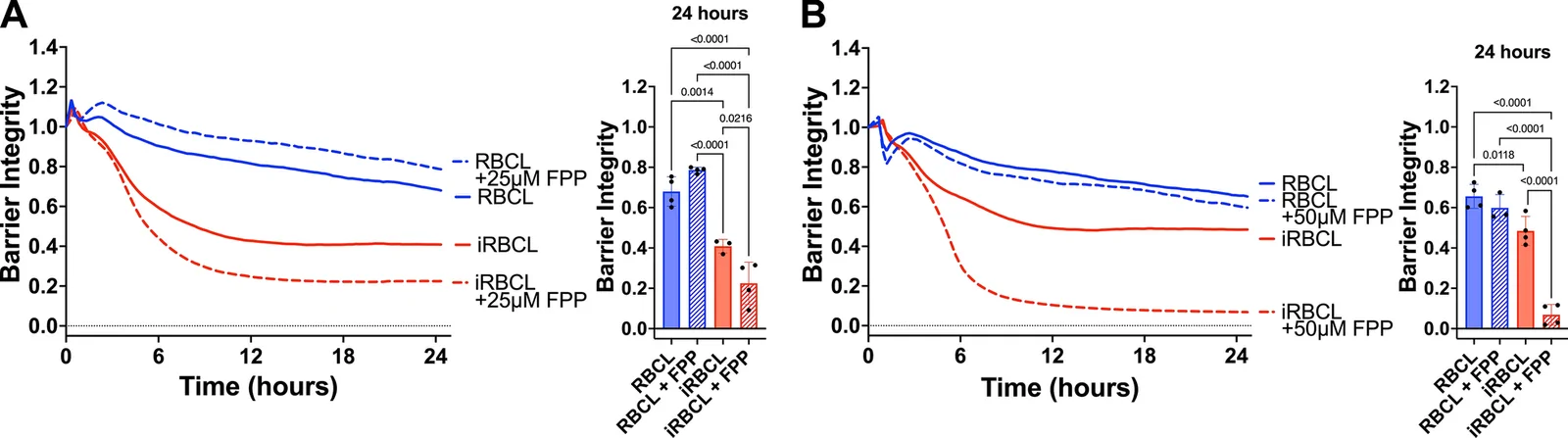
Farnesyl pyrophosphate increases HBMEC barrier permeability induced by *P. falciparum* iRBCs. 25 µM (**A**) or 50 µM (**B**) farnesyl-PP (FPP) was added to a monolayer of immortalized HBMECs 5 min before the addition of lysates of RBCs (RBCL) or iRBCs (iRBCL). Barrier integrity of HBMEC monolayers was measured by xCELLigence as impedance every 15 min over 24 h. Bars represent barrier integrity at 24 h. Results represent the average from 2 independent experiments performed in duplicate with standard deviations. Statistical significance was determined by one-way ANOVA with Tukey’s multiple comparisons test. Significant *P* values are displayed on the graphs

We first added the isoprenoid farnesyl-PP to HBMECs in combination with either *P. falciparum*-iRBCL, which is known to induce strong barrier disruption [9], or control RBCL. We observed that farnesyl-PP does not modulate HBMEC barrier integrity when added in combination with control RBCL, but supplementation of iRBCL with this lipid increases the disruption of the endothelial barrier in a dose-dependent manner (Fig. 1), suggesting a possible role for prenylation in the regulation of barrier integrity by *P. falciparum*. Indeed, a previous study showed that incubation of HBMECs with iRBCL results in a strong downregulation of multiple upstream enzymes in the isoprenoid synthesis pathway [9].

### Inhibition of prenylation protects brain endothelial cell barrier integrity from disruption caused by *P. falciparum*

**Fig. 2:**
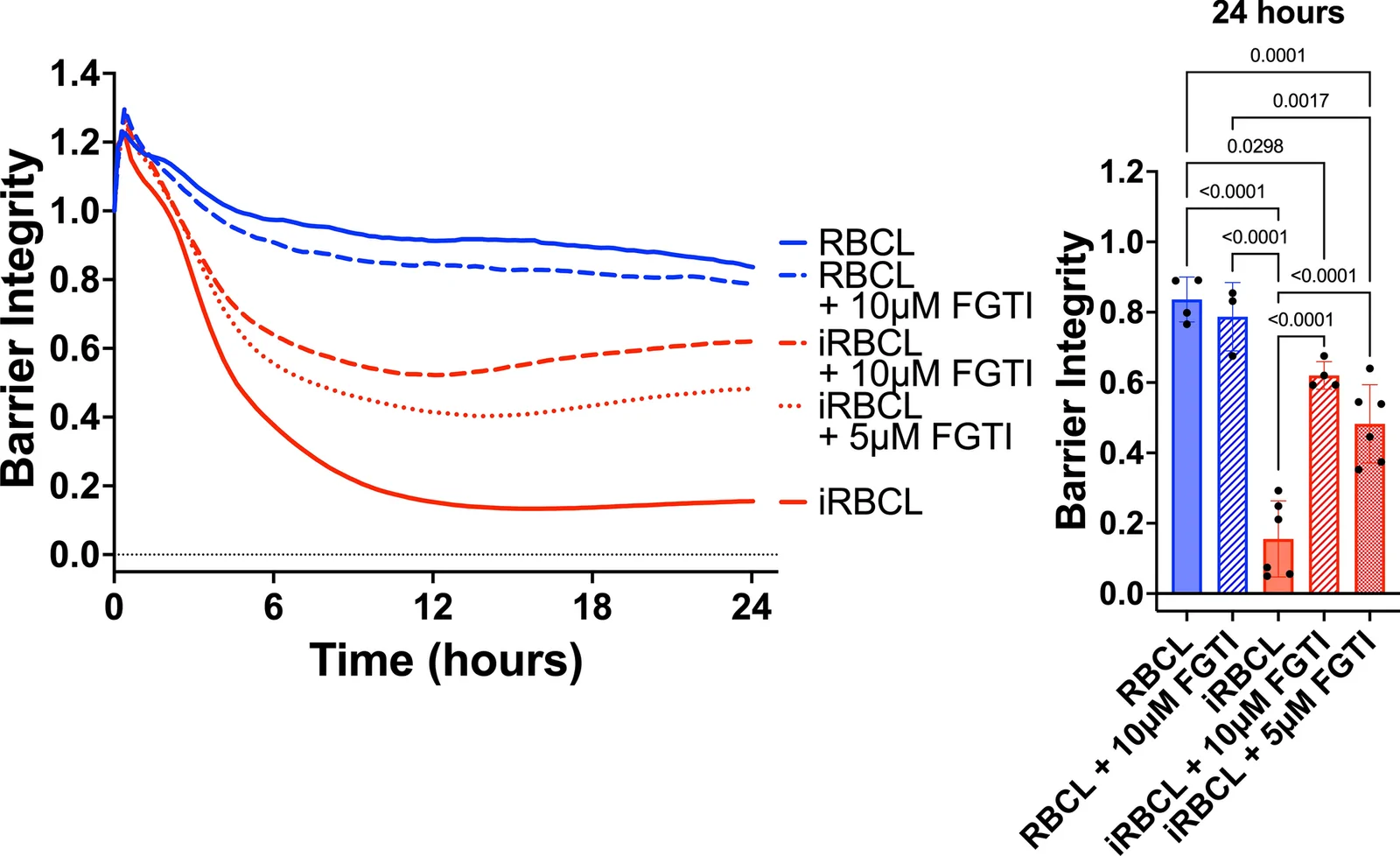
A general inhibitor of prenylation partially protects HBMEC barrier from disruption caused by *P. falciparum*-iRBCs. A monolayer of HBMECs was incubated with 5 or 10 µM FGTI 2734 (FGTI) for 1 h before adding RBCL or iRBCL. Barrier integrity of HBMEC monolayers was measured by xCELLigence as impedance every 15 min over 24 h. Bars represent barrier integrity at 24 h. Results represent the average from 3 independent experiments performed in duplicate with standard deviations. Statistical significance was determined by one-way ANOVA with Tukey’s multiple comparisons test. Significant *P* values are displayed on the graphs

To determine whether modulation of prenylation in endothelial cells by *P. falciparum* is related to its endothelial barrier disrupting activity, we first used a chemical inhibitor (FGTI) which inhibits prenylation in endothelial cells as it targets both farnesyltransferase and geranylgeranyltransferase [15]. We observed that FGTI induced partial protection of the endothelial barrier (Fig. 2), suggesting that protein prenylation modulates barrier integrity disruption caused by *P. falciparum*.

### Inhibition of farnesyltransferase, but not of geranylgeranyltransferase, protects brain endothelial cell barrier integrity from *P. falciparum*-induced disruption

**Fig. 3:**
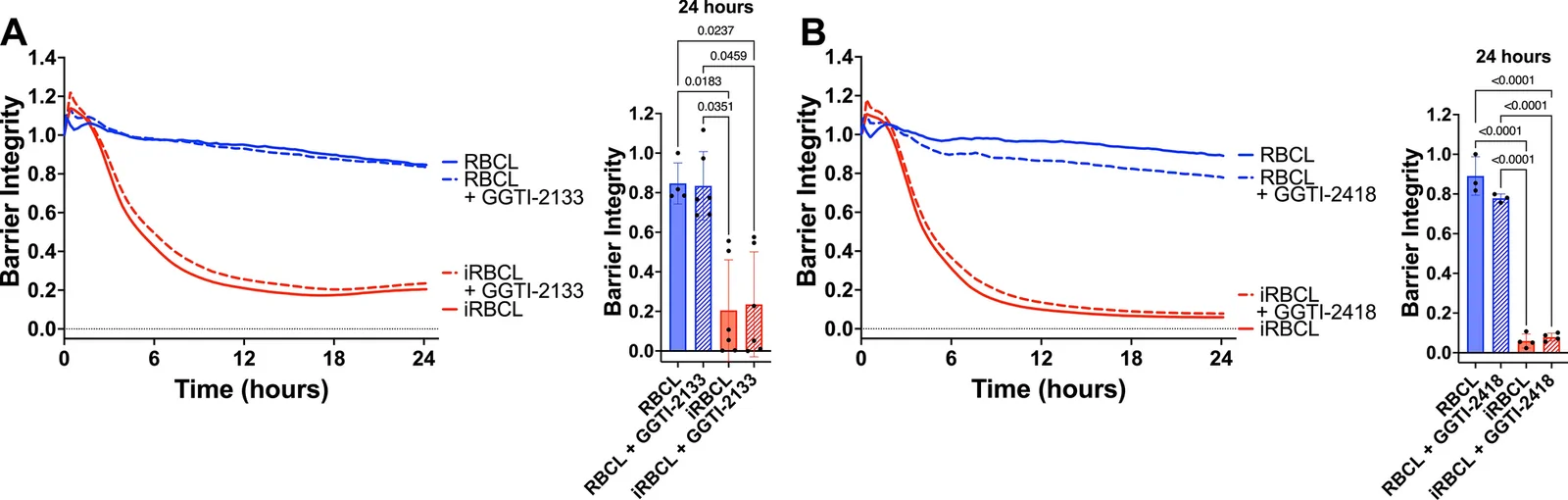
Inhibitors of geranylgeranyltransferase do not protect HBMEC barrier integrity from *P. falciparum*-iRBCs. A monolayer of HBMECs was incubated with 10 µM GGTI-2133 (**A**) or 50 µM GGTI-2418 (**B**) for 1 h before adding RBCL or iRBCL. Barrier integrity of HBMEC monolayers was measured by xCELLigence as impedance every 15 min over 24 h. Bars represent barrier integrity at 24 h. Results represent the average of at least 2 independent experiments performed in duplicate with standard deviations. Statistical significance was determined by Kruskal–Wallis test with Dunn’s multiple comparisons test (**A**) or one-way ANOVA with Tukey’s multiple comparisons test (**B**). Significant *P* values are displayed on the graphs

We next aimed to independently inhibit the two enzymes performing prenylation in endothelial cells: geranylgeranyltransferase, which incorporates geranylgeranyl, and farnesyltransferase, which incorporates farnesyl [20]. We first observed that well-characterized inhibitors of geranylgeranyltransferase, GGTI-2133 and GGTI-2418 [14, 18], did not protect HBMECs from *P. falciparum*-iRBCL induced disruption (Fig. 3).

**Fig. 4:**
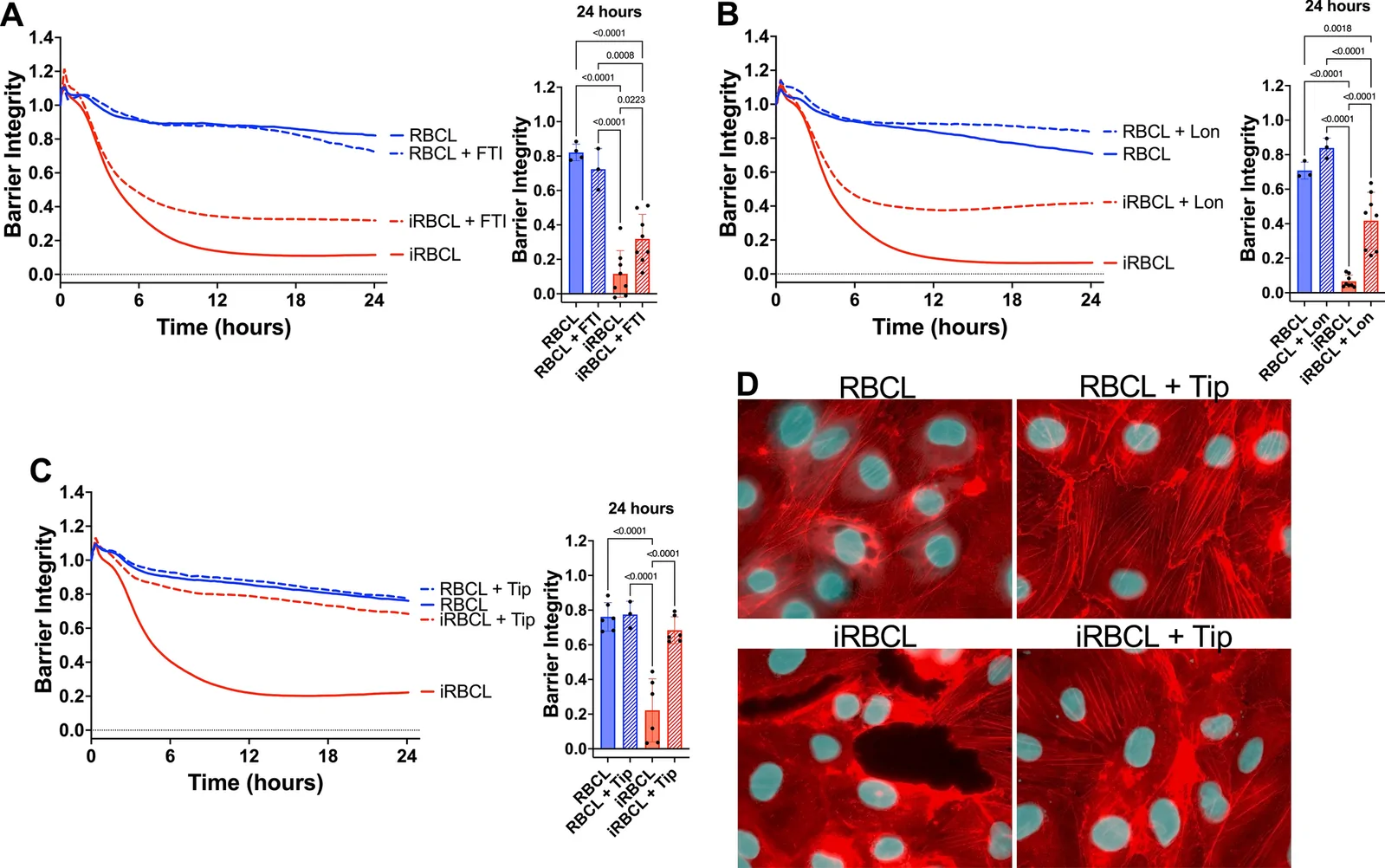
Inhibitors of farnesyltransferase protect HBMEC barrier from disruption caused by *P. falciparum-*iRBCs. A monolayer of HBMECs was incubated with 50 µM FTI 277 (FTI, **A**), 5 µM lonafarnib (Lon, **B**), or 1 µM tipifarnib (Tip, **C-D**) for 1 h before adding RBCL or iRBCL. **A**–**C** Barrier integrity of HBMEC monolayers was measured by xCELLigence as impedance every 15 min over 24 h. Bars represent barrier integrity at 24 h. Results represent the average from 3–4 independent experiments performed in duplicate with standard deviations. Statistical significance was determined by one-way ANOVA with Tukey’s multiple comparisons test. Significant *P* values are displayed on the graphs. **D** Representative images of media- or tipifarnib-treated monolayers of HBMECs that were incubated for 4 h with RBCL or iRBCL before fixation and actin (Phalloidin; red) and nuclei (Hoechst; cyan) staining

Conversely, we observed that well-characterized inhibitors of farnesyltransferase; FTI (Fig. 4A) [17], lonafarnib (Fig. 4B) [13], and tipifarnib (Fig. 4C, D) [16] induce protection of the endothelial barrier from disruption induced by *P. falciparum*-iRBCL, pointing to the importance of farnesyltransferase in this process. Notably, pretreatment with tipifarnib fully protected the HBMEC monolayer from iRBCL-induced disruption as shown by a prevention in both the decrease of electrical impedance (Fig. 4C) and gaps between the cells as shown by fluorescent microscopy (Fig. 4D).

### Tipifarnib protects barrier integrity of other endothelial cell types from *P. falciparum*-induced disruption

**Fig. 5:**
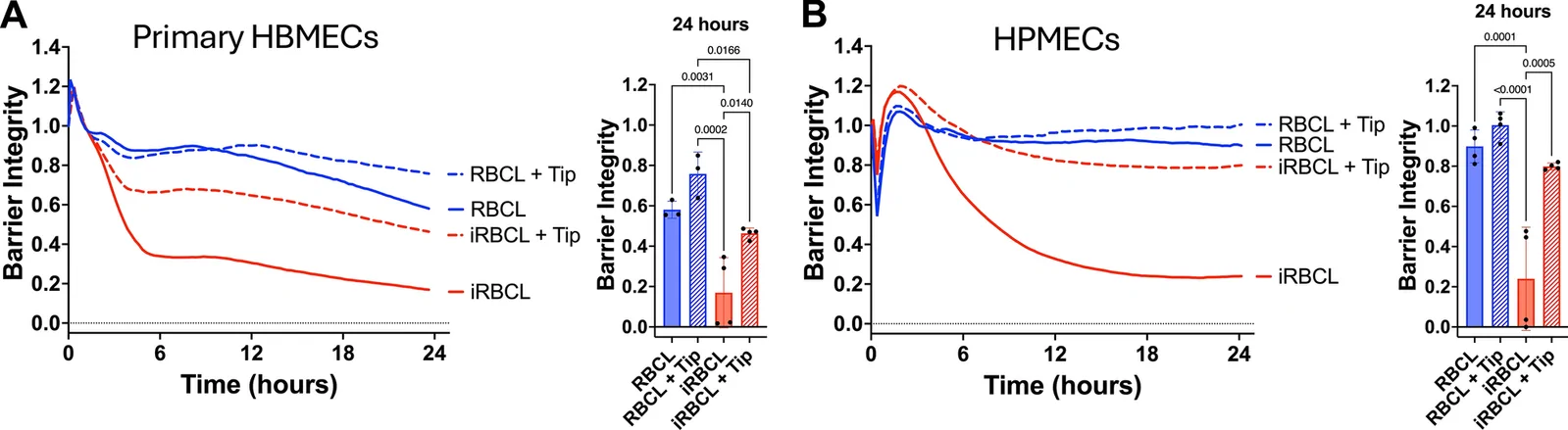
Tipifarnib protects monolayers of other endothelial cells. 1 µM tipifarnib (Tip) was added to a monolayer of primary HBMECs (**A**) or immortalized pulmonary microvascular endothelial cells (HPMECs, **B**) for 1 h before adding RBCL or iRBCL. Barrier integrity of endothelial monolayers was measured by xCELLigence as impedance every 15 min over 24 h. Bars represent barrier integrity at 24 h. Results represent the average from 2 independent experiments performed in duplicate with standard deviations. Statistical significance was determined by one-way ANOVA with Tukey’s multiple comparisons test. Significant *P* values are displayed on the graphs

Since tipifarnib induces complete protection of endothelial barrier integrity in immortalized HBMECs, we also tested this drug in primary HBMECs (Fig. 5A), as well as in immortalized human pulmonary microvascular endothelial cell (HPMECs; Fig. 5B), where it induces strong protection against the *P. falciparum-*iRBCL in both cell types.

### Tipifarnib does not protect brain endothelial cell barrier integrity against other known inducers of endothelial barrier disruption

**Fig. 6:**
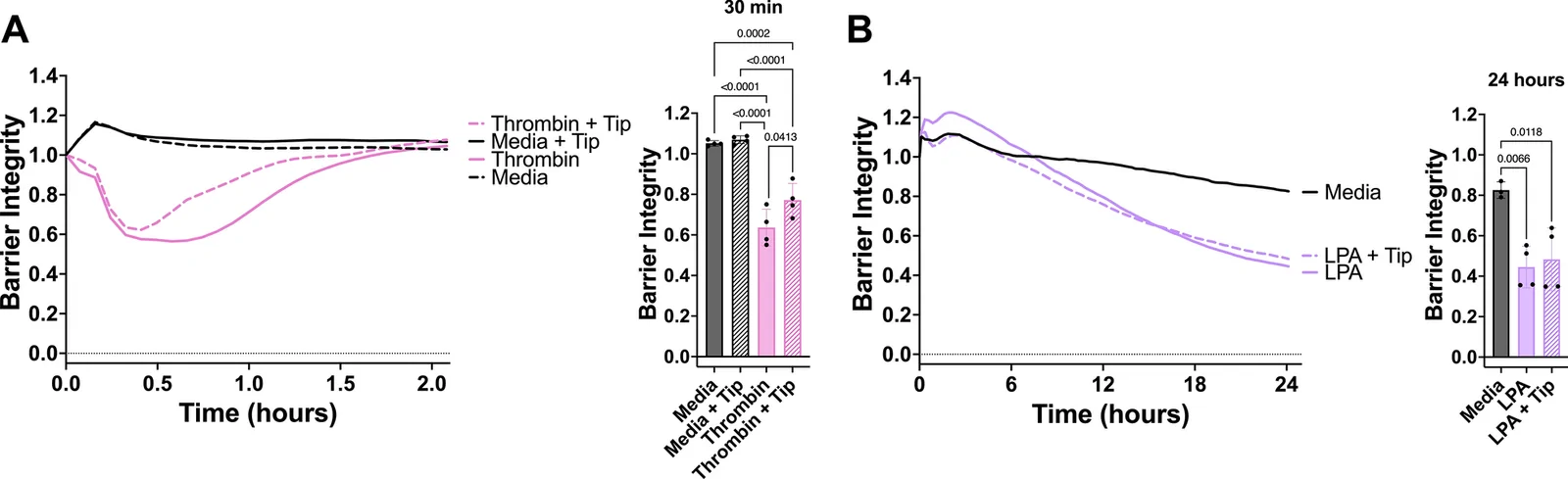
Tipifarnib partially protects HBMEC barrier integrity from disruption caused by thrombin, but not lysophosphatidic acid. HBMECs were treated with 1 µM tipifarnib (Tip) for 1 h before the addition of 1U/mL thrombin (**A**) or 25 µM 18:1 lysophosphatidic acid (LPA, **B**). Barrier integrity of HBMEC monolayers was measured by xCELLigence as impedance every 5 min over 2 h (**A**) or 15 min over 24 h (**B**) and is displayed as a representative graph (**A**) or the average of 2 experiments (**B**). Bars represent barrier integrity at indicated timepoint and the average from 2 independent experiments performed in duplicate with standard deviations. Statistical significance was determined by one-way ANOVA with Tukey’s multiple comparisons test. Significant *P* values are displayed on the graphs

Thrombin is a central enzyme in blood coagulation that is thought to contribute to endothelial barrier disruption by *P. falciparum*-iRBCs [21]. We observed that tipifarnib does not protect barrier integrity against disruption induced by thrombin, although it induces a faster recovery of the barrier integrity in immortalized HBMECs (Fig. 6A). Additionally, tipifarnib does not protect against disruption induced by lysophosphatidic acid (LPA) (Fig. 6B), a phospholipid known to disrupt brain endothelial cell barriers [22]. Taken together, these results indicate that tipifarnib does not protect the HBMEC barrier from disruption induced by *P. falciparum-*iRBCL by simply strengthening the inter-endothelial junctions.

### Treatment of mice with tipifarnib results in increased survival from experimental cerebral malaria

To determine the efficacy of tipifarnib for cerebral malaria in vivo, we used the well-established model of C57/B6 mice infection with *P. berghei*-ANKA [23]. Although this mouse model does not reflect the cytoadhesion of *P. falciparum*-iRBCs to brain endothelium, it is particularly useful to assess the effects of treatments that modulate barrier integrity [24].

**Fig. 7:**
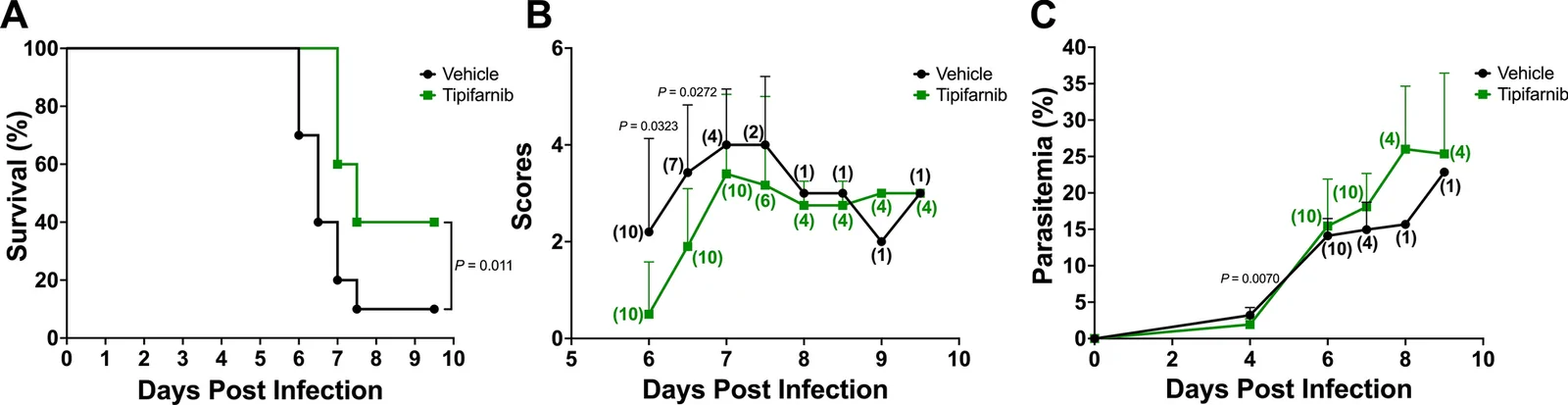
Tipifarnib increases survival in an experimental cerebral malaria mouse model. Groups of 10 mice were treated with tipifarnib 100 mg/kg or vehicle twice daily from day one before infection. **A** Statistical significance of survival was determined by Logrank (Mantel-Cox) test. **B** Cerebral malaria scores quantify coma and behavior on a scale from 0 to 5. Statistical significance was determined by Mann–Whitney test. **C** A significant difference in parasitemia was only observed on Day 4 as determined by Welch’s t test. **B**, **C** Number of surviving mice each day is indicated in parenthesis. **A**–**C** Significant *P* values are displayed on the graphs

Groups of mice were treated twice per day with tipifarnib or vehicle control until the end of the experiment, starting one day before infection with *P. berghei*. We observed that tipifarnib-treated mice presented with a delayed onset of experimental cerebral malaria signs and increased survival rates compared to vehicle-treated mice (Fig. 7A, B), while presenting equal levels of parasitemia when signs start on day 6 (Fig. 7C). These results indicate a partial protective effect of tipifarnib in experimental cerebral malaria.

## Discussion

The treatment of cerebral malaria is crucial for survival since untreated cases are nearly 100% fatal. However, treated cases still have a 15–20% mortality rate, and survivors frequently present with permanent neurological sequelae [3]. Current treatment is based on rapid intravenous administration of anti-malarial drugs [25], aiming to eliminate *P. falciparum* infection before the integrity of the BBB is compromised; nevertheless, this objective is not always achieved. Host brain endothelial cells have been proposed as a therapeutic target for cerebral malaria since treatments that preserve BBB integrity could ‘buy time’ while conventional anti-*Plasmodium* drugs eliminate the parasite, but no adjunctive treatment for cerebral malaria is currently available [5].

Our finding in this study that inhibition of farnesyltransferase has a protective effect in human endothelial cells in vitro and in a mouse model of cerebral malaria points to this enzyme as a potential target for specific therapies directed at maintaining barrier integrity during cerebral malaria. Furthermore, the protective effects of tipifarnib from *P. falciparum*-induced disruption of the endothelial barrier were also observed in pulmonary endothelial cells, suggesting that inhibition of farnesylation may also alleviate other severe malaria complications, such as respiratory distress, where different types of endothelial cells are involved.

We observed that exogenous farnesyl-PP increased the endothelial barrier disruption by *P. falciparum*, which is in line with previous findings that endothelial barrier integrity is regulated by farnesylation [10]. In the brain capillaries during cerebral malaria, where a dense accumulation of both infected and uninfected RBCs impedes blood flow [2], the rupture and release of *P. falciparum*-iRBCs at the end of their cycle must result in the accumulation of isoprenoids, including farnesyl-PP, which are very abundant in the iRBC [26]. Since farnesyl-PP is known to penetrate the cell membrane, being metabolically active [27, 28], the increase of *P. falciparum*-derived farnesyl-PP in brain endothelial cells would result in the down-regulation of farnesyl-PP synthesis pathway, which was observed in vitro after incubation of endothelial cells with *P. falciparum*-iRBCL [9].

Multiple inhibitors of farnesyltransferase protected endothelial cells from barrier disruption induced by *P. falciparum*-iRBCs, but not thrombin or LPA, suggesting that farnesylation mediates the loss of endothelial barrier integrity induced by the parasite, while thrombin and LPA disrupt through different mechanisms. In this case, the farnesyltransferase inhibitor tipifarnib would protect the endothelial barrier integrity from farnesylation-specific disrupting agents, but not others such as LPA or thrombin.

This phenomenon appears to be specific, since the inhibitors of geranygeranylation did not protect the barrier, which is compatible with the high selectivity of these post-translational modifications in their regulatory function of different cellular processes in endothelial cells [29]. Taken together, these results indicate that farnesylation regulates the disruption of the brain endothelial barrier by *P. falciparum*.

The mouse model of cerebral malaria does not fully reproduce the human syndrome since cytoadherence of human and mouse iRBCs is mediated by different adhesion molecules [30], however it is representative of the host inflammatory environment, endothelial disruption, and brain damage induced in human cerebral malaria [31]. Partial protection from experimental cerebral malaria was observed after treatment with tipifarnib in the mouse model, which is in agreement with the in vitro observations in human cells, pointing to inhibition of farnesylation as a protective treatment to increase endothelial barrier integrity before cerebral malaria. However, it is important to highlight the limitations of this in vivo study, which should be validated with an independent experiment including higher numbers of mice to increase the statistical power. In parallel, blood–brain barrier permeability should be assessed to verify the mechanism of action of tipifarnib in vivo. Additionally, the use of tipifarnib as an adjunctive therapy to an antimalarial drug, such as artemisinin, should also be explored.

Two of the inhibitors of farnesyltransferase tested in this study, lonafarnib and tipifarnib, have been developed for the treatment of human cancer. Lonafarnib is already approved for use in children and adults, while tipifarnib has been designated as a breakthrough therapy by the FDA [32]. Tipifarnib, the most effective in vitro treatment in this study, was originally developed for cancer and presents various side effects, most notably hematological complications like neutropenia and thrombocytopenia [33], that would be of concern in severe malaria patients. However, these side effects were observed in a fraction of cancer patients treated during an extended period of time (28-days) and only for specific cancer types [33]. While treatment in the context of *P. falciparum* severe malaria would be shorter (7–10 days) [34], the potential side effects of tipifarnib treatment remain unknown.

The results presented here provide evidence that farnesyl transferase inhibitors protect the brain endothelium against disruption induced by *P. falciparum* and highlight their therapeutic potential as adjunctive treatments for cerebral malaria.

## Conclusions

This study identified that inhibitors of farnesyltransferase protect the barrier integrity of different endothelial cell types from disruption caused by *P. falciparum* in vitro and decrease mortality in mice with experimental cerebral malaria. These findings indicate a role for farnesylation in the regulation of barrier integrity during cerebral malaria and identify the potential of farnesyltransferase inhibitors as adjunctive therapies in the treatment of cerebral malaria.

## Data Availability

All data generated are included in this published article. The dataset analyzed during the current study is available in the Gene Expression Omnibus (GEO) database, accession no. [GSE211439] (https://www.ncbi.nlm.nih.gov/geo/query/acc.cgi?acc=GSE211439) [9].

## Abbreviations

BBB: Blood–brain barrier
Farnesyl-PP: Farnesyl pyrophosphate
GEO: Gene Expression Omnibus
HBMECs: Human brain microvascular endothelial cells
HPMECs: Human pulmonary microvascular endothelial cells
IACUC: Institutional Animal Care and Use Committee
iRBCL: Infected red blood cell lysate
iRBCs: Infected red blood cells
RBCL: Red blood cell lysate
RBCs: Red blood cells
